# Comparative genome and transcriptome analyses of the social amoeba *Acytostelium subglobosum* that accomplishes multicellular development without germ-soma differentiation

**DOI:** 10.1186/s12864-015-1278-x

**Published:** 2015-02-14

**Authors:** Hideko Urushihara, Hidekazu Kuwayama, Kensuke Fukuhara, Takehiko Itoh, Hiroshi Kagoshima, Tadasu Shin-I, Atsushi Toyoda, Kazuyo Ohishi, Tateaki Taniguchi, Hideki Noguchi, Yoko Kuroki, Takashi Hata, Kyoko Uchi, Kurato Mohri, Jason S King, Robert H Insall, Yuji Kohara, Asao Fujiyama

**Affiliations:** Faculty of Life and Environmental Sciences, University of Tsukuba, 1-1-1 Tennodai, Tsukuba, Ibaraki 305-8572 Japan; Tokyo Institute of Technology, Yokohama, Japan; National Institute of Genetics, Mishima, Japan; Mitsubishi Research Institute, Tokyo, Japan; RIKEN Advanced Science Institute, Yokohama, Japan; Beatson Institute for Cancer Research, Glasgow, UK; National Institute of Informatics, Tokyo, Japan

**Keywords:** Multicellular development, Cell differentiation, Signaling cascade, Gene expression, Evolution

## Abstract

**Background:**

Social amoebae are lower eukaryotes that inhabit the soil. They are characterized by the construction of a starvation-induced multicellular fruiting body with a spore ball and supportive stalk. In most species, the stalk is filled with motile stalk cells, as represented by the model organism *Dictyostelium discoideum*, whose developmental mechanisms have been well characterized. However, in the genus *Acytostelium*, the stalk is acellular and all aggregated cells become spores. Phylogenetic analyses have shown that it is not an ancestral genus but has lost the ability to undergo cell differentiation.

**Results:**

We performed genome and transcriptome analyses of *Acytostelium subglobosum* and compared our findings to other available dictyostelid genome data. Although *A. subglobosum* adopts a qualitatively different developmental program from other dictyostelids, its gene repertoire was largely conserved. Yet, families of polyketide synthase and extracellular matrix proteins have not expanded and a serine protease and ABC transporter B family gene, *tagA*, and a few other developmental genes are missing in the *A. subglobosum* lineage. Temporal gene expression patterns are astonishingly dissimilar from those of *D. discoideum*, and only a limited fraction of the ortholog pairs shared the same expression patterns, so that some signaling cascades for development seem to be disabled in *A. subglobosum*.

**Conclusions:**

The absence of the ability to undergo cell differentiation in *Acytostelium* is accompanied by a small change in coding potential and extensive alterations in gene expression patterns.

**Electronic supplementary material:**

The online version of this article (doi:10.1186/s12864-015-1278-x) contains supplementary material, which is available to authorized users.

## Background

Morphogenesis and cell differentiation are the major components of multicellular development. In multicellular organisms, somatic cells, which are free from regenerative obligations, accomplish a variety of tasks to support complex body structures and functional integrity. The differentiation of mortal or sacrificial somatic cells from reproductive germ cells was the key event for the establishment and diversification of multicellular systems. How this was achieved in the history of life is an interesting and complex issue [1,2].

The social amoebae are unique organisms that exhibit conditional multicellularity and serve as an excellent model system to address this issue; they grow as solitary amoeba in the presence of sufficient food, but when starved, they gather together and form a multicellular fruiting body composed of a spore ball(s) and a supportive stalk(s). In many species, the stalk is filled with vacuolated stalk cells to stiffen it using osmotic pressure and cellulose walls that are deposited and polymerized on the extracellular matrix (ECM). While spores transmit their genetic information to their offspring, the stalk cells are no longer regenerative and represent one of the simplest forms of terminally differentiated somatic cells. In *Dictyostelium discoideum*, the most widely analyzed social amoeba species, cells in the migratory slug are committed to either the spore (prespore cells) or stalk lineage (prestalk cells) [3]. The latter further diversifies to generate the prestalk subpopulations PstB, PstO, and PstU, which end up in the basal disk and upper and lower cup structures, in addition to PstA constituting the main stalk body. These developmental processes are mainly controlled by the external levels of chemical cues such as cyclic nucleotides, ammonia, polyketides, peptides, and steroids to activate the corresponding intracellular signaling cascades [4].

**Figure 1:**
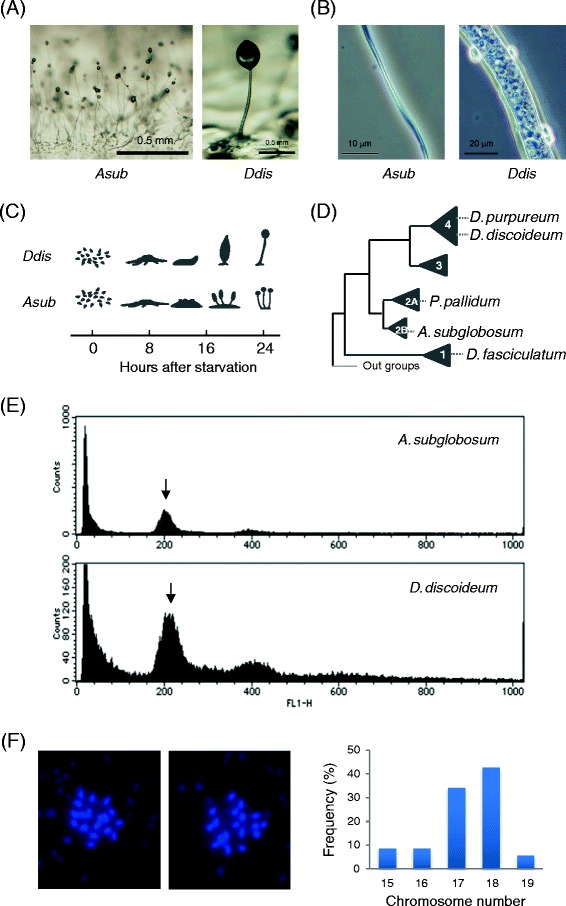
**Properties of** ***A. subglobosum*** **in comparison with** ***D. discoideum.*** **A**: Morphologies of fruiting bodies of *A. subglobosum* (left) and *D. discoideum* (right). Note the differences in magnification. **B**: Higher magnification photographs of *A. subglobosum* (left) and *D. discoideum* stalk (right). **C**: Developmental time courses for both species. **D**: Phylogenetic relationships shown schematically for the species described in the text. Numerals in the triangles indicate the group number of each clade. **E**: Results of flow cytometry analysis of nuclear DNA content. Arrows indicate the peak positions of the haploid nuclei. **F**: DAPI staining of the nuclei. Two independent nuclei (left pictures) and sum of 35 nuclei (right) are shown.

In spite of the common nature of starvation-induced fruiting body formation, species in the genus *Acytostelium* form an acellular stalk [5] and all aggregated amoebae become spores [6] (Figure 1A, B). They skip the migratory stage and multiple tips arise directly from the cell aggregates to generate crown-type fruiting bodies (Figure 1C). Recently, we studied the development of *Acytostelium subglobosum* and found that the aggregated cells first became prespore-like cells producing spore coat proteins, and then function as if they were prestalk cells to synthesize and secrete the ECM and cellulose, and finally achieve terminal differentiation of the spores at the top of culminants [6]. Thus, temporal but not permanent division of labour is observed in this species.

According to molecular phylogenetic analyses, *D. discoideum* belongs to the newest evolutionary clade (group 4), while the genus *Acytostelium* is in an older clade (group 2) [7,8] (Figure 1D). There are two possibilities for the lack of the ability to undergo cell differentiation in *Acytostelium*: this ability was either acquired in a later species or it had been acquired in a common ancestor and lost in the *Acytostelium* lineage. Since the species in the oldest clade, group 1, form fruiting bodies with cellular stalks, the latter possibility seems more likely, although it is still possible that cellular stalks arose independently multiple times, as pointed out by Swanson *et al*. [9]. In either case, it is intriguing to determine what genetic information correlates with the ability to undergo germ-soma differentiation.

In the present study, we analyzed the genome and transcriptome of *A. subglobosum* in comparison with other social amoebae making cellular stalks. The *D. discoideum* genome was reported initially in 2005 [10]. Since then, the genomes of *Dictyostelium purpureum* (group 4) [11], *Polysphondylium pallidum* (group 2), and *Dictyostelium fasciculatum* (group 1) [12] have become available. The developmental transcriptome of *D. purpureum* was compared with that of *D. discoideum* to reveal their remarkable conservation [13]. Our comparative analyses showed that dissimilarities in the gene repertoire between differentiating and non-differentiating species were limited, but that their transcriptomes had diverged. We suppose that the critical loss of early developmental genes relevant to cell-type specification affected the gene networks and led to the invention of a new developmental program where the entry of the entire amoeba to germ-line spores was traded off against the low efficiency of their dispersal due to short and fragile acellular stalks that were unable to support sizable spore balls*.*

## Results and discussion

### Structure and general features of the *A. subglobosum* genome

The genome of *A. subglobosum* LB-1/A1 was sequenced using a whole genome shotgun sequencing approach and assembled into 371 contigs (DDBJ:BAUZ01000001-BAUZ01000371) arranged into 106 supercontigs (Table 1, Additional file 1: Figure S1). The total extension of the nucleotide sequence was 30.9 Mbp and close to the size of *D. fasciculatum*, the smallest dictyostelid genome analyzed so far. Since there was no available information on the genome size of *A. subglobosum*, we used comparative flow cytometry analysis of nuclear DNA content to estimate its size as approximately 29 Mbp (Figure 1E). Although there is a small discrepancy between the two numbers, we assume that the present data adequately represent the *A. subglobosum* genome. The chromosome number is 18 (Figure 1F). This is much larger than in the other dictyostelid species, but the possibility that it is diploid was shown to be unlikely from the results of flow cytometry. The (A + T) content of the *A. subglobosum* genome is 55%, which is remarkably lower than the other dictyostelid species and naturally results in differential codon usage. It is much less biased compared to that of *D. discoideum* (Additional file 2: Table S1). The rRNA genes were found clustered in one of the supercontigs (SC 64). The number of tRNA genes was the smallest among the analyzed species. Simple sequence repeats and transposons were also not abundant. As a whole, our sequencing data revealed the neutral base composition, compact nature, and rather static features of the *A. subglobosum* genome.

**Table 1:** **General features of** ***A. subglobosum*** **and other dictyostelid genomes**

	***Asub***	***Ddis***	***Dpur***	***Ppal***	***Dfas***
Phylogeny group	2	4	4	2	1
Genome size (Mbp)^a^	31	34	33	33	31
Chromosome number	18	6		7	6
Supercontigs	106	6	799	41	25
Contigs	371	226	1,213	52	33
Genome (A + T) content (%)	55.0	77.6		68.0	66.2
Protein coding genes	12722^b^	13213	12410	12373	12173
Gene density (CDS/Mbp)	410	396	376	375	392
tRNAs	167^c^	390		273	198
Simple sequence repeat (%)	3.8	11.0	4.4		
DNA transposons	1	215			
LTR transposons	13	315	1.1 Mb		
Non-LTR transposons	57	235			
Reference	This work	[10]	[11]	[12]	[12]

^a^Total extension.

^b^Only proteins predicted to be >50 amino acids were counted.

^c^Results of stand-alone tRNAscan-SE [14].

### Protein coding potential of *A. subglobosum*

We performed expressed sequence tag (EST) analysis of vegetative and developmental cDNA libraries to determine the protein coding potential of *A. subglobosum*. Altogether, 32000 clones were read from both ends (DDBJ:HY448297-HY508708), and the obtained sequences were clustered into 7439 non-redundant groups derived from 5749 genes, 98.4% of which were successfully mapped to the genome at an identity ≥ 95% and coverage ≥ 80%. Representative cDNA clones were chosen for each of these groups and re-sequenced. The transcript information thus obtained (Additional file 1: Figure S2) was incorporated into a gene prediction program based on dicodon analysis [15] (Additional file 1: Figure S3). We also constructed homology-based gene models using *D. discoideum* protein sequences. The three sets of gene models, actual transcript sequences, *ab initio* predictions, and homology based predictions were combined to generate 12722 non-redundant protein coding genes (Additional file 2: Table S2; Additional file 3). This gene number is smaller than *D. discoideum*, but larger than 3 other species (Table 1). Lack of stalk cells in *A. subglobosum* may therefore not be explained simply by the absence of gene sets required for cell differentiation and stalk-cell function. This supports the previous argument that the ability to undergo cell differentiation was lost in *A. subglobosum* rather than its acquisition in later species.

### Orthology and gene family analyses

**Figure 2:**
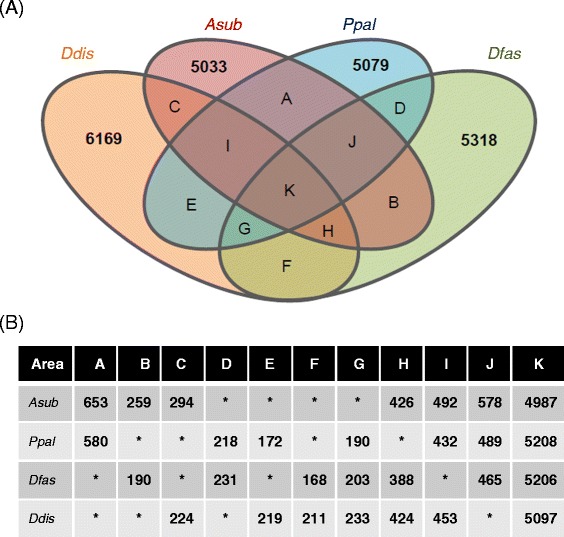
**Gene orthology analysis of 4 social amoeba species. A**: Results of the bidirectional best hit approach are shown schematically. Numbers represent species-specific genes. Colors orange, red, blue, and green represent *D. discoideum*, *A. subglobosum*, *P. pallidum*, and *D. fasciculatum*, respectively. Genes in area G are absent in the *A. subglobosum* lineage but present in *D. discoideum*, *P. pallidum*, and *D. fasciculatum*. **B**: Gene numbers of each species contained in the areas A–K of the Venn diagram.

The gene orthology relationships were analyzed by the standard bidirectional best hit approach. When the e-value threshold was set to 1E-10, 4987 *A. subglobosum* genes were shared as orthologs with 3 other species (Figure 2, area K), but 190 *P. pallidum*, 203 *D. fasciculatum*, and 233 *D. discoideum* genes had no orthologs in the *A. subglobosum* lineage (Figure 2, area G). Heidel et al. [12] took a more conscientious approach by employing identity and coverage factors and described that 6569 *D. discoideum* genes were shared with *P. pallidum* and *D. fasciculatum*. The corresponding gene number was 5330 in our analysis (sum of K and G in Figure 2). Since changing the threshold value did not result in an increased number of orthologs, we did not change the above number and examined the genes of interest individually in the later analyses.

To analyze the lineage-specific expansion of gene families in *D. discoideum*, we combined the homology search results and OrthoMCL [16] data of *D. discoideum*. Namely, homologous genes for each *D. discoideum* gene were assigned to the same gene family. Clustering of *D. discoideum* proteins by OrthoMCL resulted in similar but slightly bigger families than those reported by Eichinger *et al*. [10] (Additional file 1: Figure S4). For the 5414 OrthoMCL OG-ids of *D. discoideum* [17], which did not exclude the unique genes in this species, 4582 *A. subglobosum*, 4468 *P. pallidum*, and 4445 *D. fasciculatum* gene families (and unique genes) were associated.

**Figure 3:**
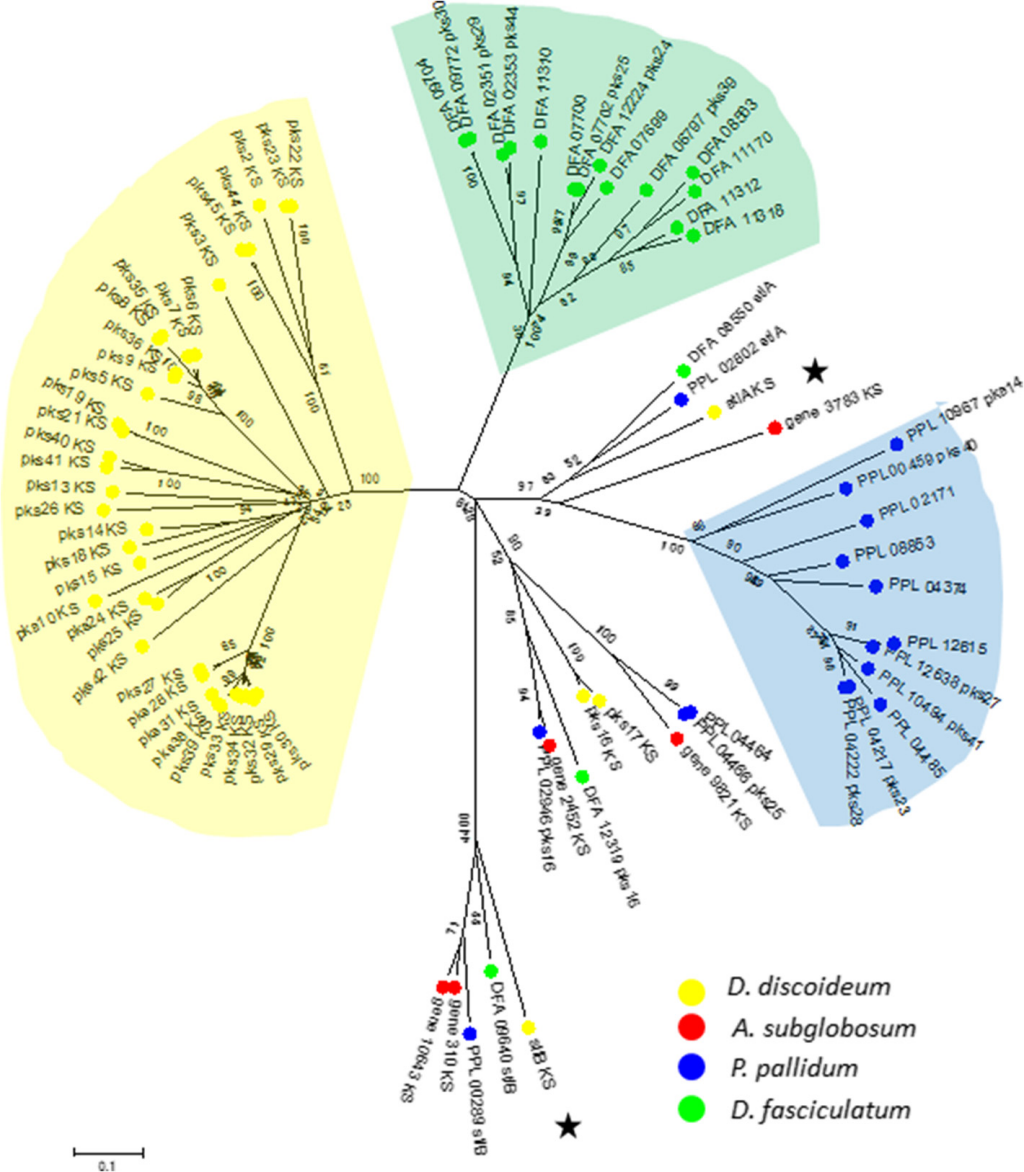
**A phylogenetic tree of the PKS family (OG5_126633) showing the** ***A. subglobosum*** **-specific lack of expansion.** Color designations are shown at the bottom right. Black stars indicate the positions of *stlA* and *stlB*.

Besides common expansions, a significant number of lineage-dependent family expansions were observed (Additional file 1: Figure S5). Notable examples of them are marked and detailed in Table 2. The polyketide synthase (PKS) family (OG5_id 126633), whose members function in the biosynthesis of diverse classes of natural products such as those involved in intercellular and ecological interactions [10,18,19], is very small in *A. subglobosum*. This suggests that these activities are less extensive in this species. Yet, *A. subglobosum* does have homologs of *stlB*, involved in the biosynthesis of differentiation inducing factor-1 (DIF-1) [20], which is necessary for the induction of stalk-cell subpopulations [21,22], and *stlA* [23,24], which is involved in the signaling cascade for spore maturation in *D. discoideum* (Figure 3). Likewise, the ECM family (OG5_133822), which is expressed predominantly in prestalk cells and is required for cellulose deposition and polymerization [25], has not expanded in *A. subglobosum*, reflecting the small and less complex structure of its fruiting bodies.

**Table 2:** **Lineage-dependent expansion of gene families**

**OG5-id**	**Description**	**Family size in**				**Mark** ^**#**^
		***Ddis***	***Asub***	***Ppal***	***Dfas***	
**Expansion in non-** ***Dfas*** **lineage**						
126643	Zinc finger, B-box domain and FNIP repeat-containing protein	241	381	206	55	a
**Expansion in non-** ***Ddis*** **lineage**						
153020	IPT/TIG domain-containing protein, EGF-like domain-containing protein, C-type lectin domain-containing protein	9	43	35	50	b
**Expansion in Group 2**						
138577	Colossin D, Cna B-type domain-containing protein	5	33	22	7	c
**Expansion in** ***A. subglobosum*** **lineage**						
181792	N-terminal delta endotoxin domain-containing protein	4	27	6	4	d
**Lack of expansion in** ***A. subglobosum***						
133822	Cellulose-binding domain-containing protein, putative extracellular matrix protein	30	4	25	18	e
126633	Putative polyketide synthase, beta-ketoacyl synthase family protein	41	6	21	25	f

^#^Marks in Additional file 1: Figure S5.

### Search for genes associated with the ability to undergo cell differentiation

The main purpose of this study was to mine out genetic information that was correlated with the ability to undergo cell differentiation. It was noted, at the early stage of the study, that the major *D. discoideum* genes related to stalk-cell differentiation were present in *A. subglobosum* despite the absence of stalk cells in this species. Although this is seemingly contradictory, it is not irrational considering the fact that *A. subglobosum* does make stalks. Apparently, a simple loss of “stalk genes” cannot explain the unique developmental process of *A. subglobosum*.

As already mentioned, 233 *D. discoideum* genes with orthologs in *P. pallidum* and *D. fasciculatum* did not have specific orthologs in the *A. subglobosum* lineage. Only 5 of them, 3 unique genes and 2 family-constituting genes, are currently known to be related to culmination and cell differentiation (Table 3). Of special interest is the *tagA* gene, which encodes a putative serine protease and ABC transporter B family protein and has been reported to play a crucial role in cell-fate determination and maintenance of the spore lineage [26]. Although there are a large number of ABC transporters in *A. subglobosum*, as in other dictyostelids, there is only 1 member (gene_6301) of the B subgroup with a serine protease domain (OG5_134947). Gene_6301 encodes a protein orthologous to TagC (Additional file 1: Figure S6) and is expressed similarly to it at the later stages of development (Additional file 2: Table S2).

**Table 3:** ***D. discoideum*** **developmental genes lacking orthologs in the** ***A. subglobosum*** **lineage** ^**a**^

**Gene** ^**b**^	**Product**	**Mutant information** ^**c**^
Involved in cell fate-determination		
*tagA* (DDB_G0293002)	ABC transporter B family protein, serine protease	Multiple tips in mound (partial KO); Aberrant fruiting body morphology (KO)
Involved in stalk-cell diversification		
rtaA* (DDB_G0271852)	Lipid-translocating exporter family protein	Expressed in PstU cells (*lacZ* fusion)
warA* (DDB_G0291075)	Putative homeobox transcription factor	Development arrests at slug stage, decreased prespore cell differentiation, and increased PstO cell differentiation (KO)
Involved in terminal differentiation		
*expl7* (DDB_G0288331)	Expansin-like protein	Aberrant culminant morphology (OE)
rsc12* (DDB_G0277871)	Unknown	Aberrant culmination (KO)

^a^Only those related to morphogenesis and cell differentiation are listed.

^b^Unique genes are asterisked. Parentheses indicate DDB_G ID.

^c^Phenotypes of disruptants (KO) and overexpressors (OE), and product localization (*lacZ* fusion) were extracted from the dictyBase phenotype summary [27].

Other genes in Table 3 are expressed at the later stages of *D. discoideum* development and their relevance to cell-fate determination is less likely. Two genes, *rtaA* and *warA*, are concerned with prestalk-cell diversification. The *rtaA* gene encodes a putative GPCR [28,29] expressed in PstU cells [30], which finally populate the upper cup of a fruiting body whose function is to lift up the spore mass [31]. The latter gene, *warA*, is a homeodomain-containing putative transcription factor expressed in prestalk cells and determines the proportion of PstO cells in the slug, which occupy the zone between the prestalk and prespore regions [32]. Since the corresponding cell populations do not exist even temporarily in *A. subglobosum*, the mechanistic aspect of culmination seems different in this species, as pointed out by Bonner [2]. The *expl7* gene encodes a member of the expansin-like protein family and is homologous to the plant cellulose-binding protein expansin [33]. Although the over-expression of *expl7* resulted in an anomaly of stalk morphology, its disruption did not affect the fruiting body morphology in *D. discoideum* [34], suggesting complementation by its paralog(s). The disruption mutant of *rsc12* resulted in aberrant culminant at the final stage of development [35], but detailed analysis has not been carried out.

Loss of function can also be caused by the acquisition of new genes with suppressive effects. Although it is possible that some such genes may act to suppress the *A. subglobosum* counterparts of developmental genes in stalk-cell-making species, their functions in relation to fruiting body formation are elusive without molecular biological analyses.

### Comparative transcriptome analysis

The above mentioned analysis on the gene repertoires demonstrated that the majority of genes involved in fruiting body formation in *D. discoideum* have orthologs in *A. subglobosum* and only few of them are without counterparts. To clarify whether the conserved genes are actually expressed, the developmental transcriptome of *A. subglobosum* was analyzed. Approximately 4.5 Gb of cDNA reads, generated at each of 0, 8, 16, and 24 h of development, were combined, clustered, and mapped to the *A. subglobosum* genome contigs (Additional file 1: Figure S7), resulting in the association of at least 9067 gene models. For the 5961 *A. subglobosum* orthologs to *D. discoideum* genes with a significant level of expression, expression data for 5062 (85%) genes were obtained. As for the developmental gene orthologs, 70 had no clear evidence of expression during growth and asexual development.

**Figure 4:**
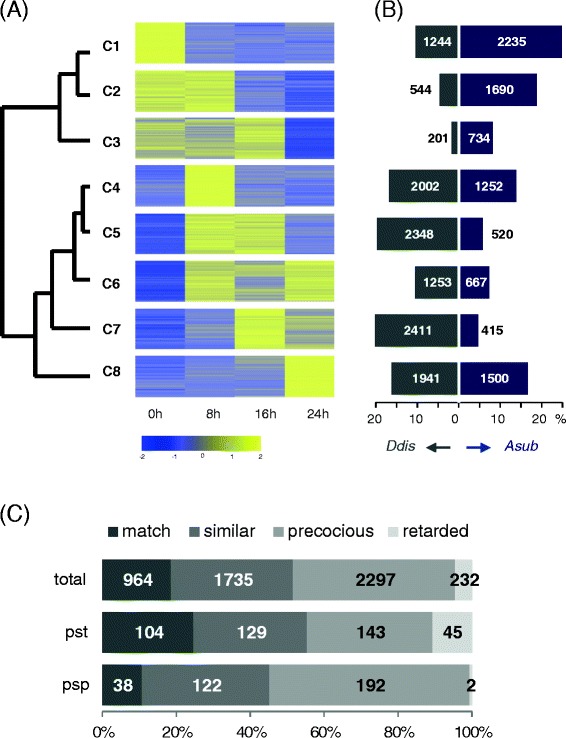
**Comparison of temporal gene expression patterns between** ***D. discoideum*** **and** ***A. subglobosum*** **. A**: Results of collective clustering are shown as heat maps with the scale shown below. Cluster distances are shown on the left. Clusters 1–3 represent down-regulated genes, while clusters 4–8 contain up-regulated genes during development. **B**: Fractions of genes in each cluster for *A. subglobosum* (right) and *D. discoideum* (left). Numbers indicate the genes in each cluster. **C**: Differential expression patterns of orthologous gene pairs for total (top), prestalk-specific (middle), and prespore-specific genes (bottom). Match: in the same cluster; similar: different cluster but in the same group; precocious: *A. subglobosum* genes were in the up-regulated group, while *D. discoideum* orthologs were in the down-regulated group; retarded: opposite of precocious.

In an attempt to compare the temporal expression patterns of orthologous pairs, the expression data of *A. subglobosum* obtained here and those of *D. discoideum* [13] were clustered collectively by K-means clustering, after normalization and standardization (Additional file 2: Table S3), using the cluster number 8 (Figure 4A). The resulting clusters fell into 2 major groups according to their mutual distances: clusters 1–3 and 4–8. The average expression pattern of the former group is down-regulation and the latter, up-regulation during development. There was a striking difference between the 2 species in gene distribution among the clusters (Figure 4B). Clusters 4 and 8 contain comparable fractions of *A. subglobosum* and *D. discoideum* genes, while the other clusters are extremely biased to either species*.* More than 50% of the analyzed genes were in the down-regulated clusters (C1–C3) in *A. subglobosum*, while less than 20% were so in *D. discoideum*. These differences are in contrast to a report on *D. purpureum* transcriptome analysis that demonstrated a remarkable conservation in gene expression patterns with *D. discoideum* [13,36], indicating the distant anatomical nature of the *A. subglobosum* fruiting body. (Additional file 1: Figure S8).

In accordance with the overall dissimilarity, only 18% of the orthologous gene pairs were assigned to the same clusters and nearly half of the ortholog pairs belonged to different groups (C1–C3 *vs.* C4–C8) (Figure 4C). As can be seen in Figure 4C, the *A. subglobosum* orthologs for prespore-specific genes, showed the stronger propensity for precocious expression, while prestalk-specific gene orthologs displayed the opposite pattern. This tendency for differential expression coincides with our previous finding that *A. subglobosum* produces prespore vesicles soon after aggregation and then makes stalk materials [6]. In consideration of development under starvation stress, it should be a safe strategy to transcribe the germ-line (spore lineage) genes first and to use the rest of the available energy for stalk formation, unless the population is divided into germ (spore) and soma (stalk cells) lineages; the stalk volume can be variable, but the amount of spore coat material is definite. The *D. discoideum* developmental genes whose orthologs belong to distant clusters are listed in Table 4. Interestingly, genes important for the terminal differentiation of spores and stalk cells in *D. discoideum* such as *acbA*, *dgcA*, and *gtaC* were expressed very early and down-regulated rapidly in *A. subglobosum* despite their presumed functions at the latest stage (Table 4). They may be translationally or post-translationally regulated or might have different functions in *A. subglobosum*.

**Table 4:** ***D. discoideum*** **developmental genes with altered expression in** ***A. subglobosum***

**Cluster #**		**Cell-type specificity in** ***D. discoideum***		
**Ddis**	**Asub**	**Prespore**	**Prestalk**	**Non-specific**
C1	0		*(nxnA)*	(4)
	C4		*gabT, proB*	aprA*, cf50-1*, cmfA, ctnA*
	C6		*scrA*	
	C8			***gpaF***
C2	0			(2)
	C4		*icmA*	*cnxA*
	C5			*psmC1*
	C8			gpaI*
C4	0		*(krsA), (paxB)*	(14)
	C1			*mgp2, mppA1, sglA, tmem184C, DG1060*
	C2		***DG1112*** , gsr, spkA*	*alg9,* ***midA*** *, ppp4C, sgcA, DG1104, DDB_G0287723*
	C3		*crtA, psmA1*	*clc, phlp1, DG1040*
C5	0			(11)
	C1		*plbG*	*hdaB, sr*
	C2	*adrm1, DG1122*	*exoc2*	cshA*, ctr9, Dd5P4, nfaA, snfA, DDB_G0278945
	C3			*kif12, lvsD, DDB_G0269680, DDB_G0285083, DDB_G0288007*
C6	0			(12)
	C1			amtA*, dymA*, srfA
	C2	*DG1124*	*fpaA*	*atg5, dgkA, DDB_G0270344*
	C3		ahhA*	
C7	0	*adprt3* *,* *tipC*	*(elmoA)*	(5)
	C2		cf60*	***captC*** *, ifkA,* ***DG1003***
	C3			*torA,* ***dnmA***
C8	0		*(mhcA)*	(6)
	C1		*dgcA*	*alrA, dokA, phyA, vmp1*
	C2	*acbA*	*gtaC*	

Genes with increased expression levels in *A. subglobosum* are shown in bold face and depressed expression levels in marked by asterisk. Parentheses indicates no expression in *A. subglobosum.* Numbers in parentheses show non-specific genes without expression during growth and development. Genes are shown by DDB_G ID in Additional file 2: Table S4. The complete comparative data are shown in Additional file 2: Table S2.

In addition to the differential expression time course, altered mRNA levels, both increased and decreased, were noticed in a substantial number of ortholog pairs (Additional file 1: Figure S9). It caught our attention that counting factor and related components involved in determination of aggregate size (*cfn50-1*, *ctnA*, and *cf60*) were greatly repressed. Only one of the related genes, *cfaD*, is expressed at a comparable level and in the same pattern, but this gene is presumed to control the growth-development transition. Since inactivation of these genes in *D. discoideum* results in larger aggregates, the implications of the above finding on the small fruiting bodies of *A. subglobosum* are unclear. It may be related to the fact that *A. subglobosum* development is possible only at lower cell densities than for *D. discoideum* development [6]. It is also interesting that the expression of genes for G protein α subunits 6 (*gpaF*) and 9 (*gpaI*) were altered in mutually opposite directions. These transcriptome differences should exert significant influences on the signal-response cascades and gene networks during development.

### Signaling cascades for cell differentiation and morphogenesis

**Figure 5:**
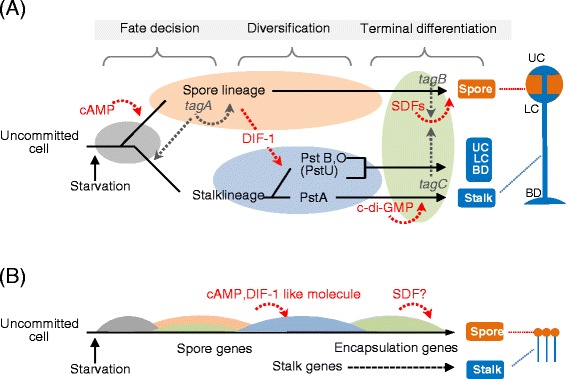
**Comparison of the overall process of fruiting formation between** ***A. subglobosum*** **and** ***D. discoideum*** **. A**: Overall process of fruiting body formation in *D. discoideum* shown schematically as gene-expression relays. Extracellular signaling molecules are shown in red. Gray, orange, blue, and green ovules represent the expression of aggregation, spore-lineage, stalk-lineage, and terminal differentiation genes, respectively. UC: Upper cup; LC: lower cup; BD: basal disk. **B**: Possible gene-expression relays in *A. subglobosum* shown in correspondence with **(A)**.

**Figure 6:**
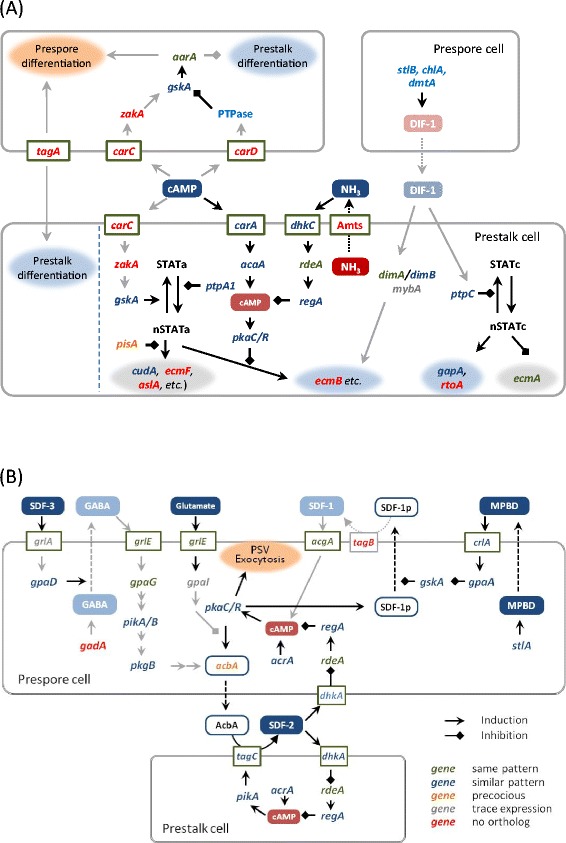
**Developmental signaling in** ***D. discoideum*** **overlaid with genomic and transcriptomics data of** ***A. subglobosum*** **. A**: Signaling cascades for cell-fate determination and prestalk differentiation in *D. discoideum*. **B**: Signaling cascades for spore encapsulation (PSV exocytosis) in *D. discoideum*. The cascades are overlaid with *A. subglobosum* genome and transcriptome information. Extracellular and intracellular signaling molecules are indicated by blue and red rectangles, respectively. Components of intracellular cascades are mostly shown by gene names but some in **(A)** are by protein names. Faint colors indicate that operation in *A. subglobosum* is unlikely. Solid arrows indicate induction or inhibition, while dotted lines indicate processing and/or transport. Genes in red designate the absence of orthologs, while those in green, blue, orange, and gray designate the same, similar, precocious, and trace expression in *A. subglobosum*, respectively. PSV: prespore vesicle.

The cell differentiation process in *D. discoideum* proceeds in 3 major steps: cell-fate determination, diversification of the prestalk cell lineage, and terminal differentiation of the spores and stalk cells (Figure 5A). To examine how genomic and transcriptomics properties of *A. subglobosum* related to these steps, the results of gene orthology and transcriptome analyses of *A. subglobosum* were overlaid on the known signaling cascades controlling *D. discoideum* development (Figure 6).

The spore lineage cells induced by a high concentration of extracellular cAMP in turn induce uncommitted cells to the prestalk lineage in *D. discoideum*. The important gene at this step, *tagA*, is missing, as mentioned above, and orthologs of two cAMP receptors, cAR3 and cAR4 (encoded by *carC* and *carD*), are also absent in *A. subglobosum*, corresponding to the lack of cell-type differentiation in this species. The specific substrate of TagA is suspected to be the acyl coenzyme A binding protein (AcbA) from the genetic evidence [37]. The facts that lack of the transporter domain of *tagA* caused the multi-tip phenotype in *D. discoideum* but that complete disruption of this gene resulted in a single gnarled stalk suggest the dual function of TagA during development.

Stalk-cell diversification is triggered by DIF-1, which is secreted from prespore cells in *D. discoideum*. Despite the fact that *A. subglobosum* does not make the basal disk or upper and lower cup structures of the fruiting body, genes for the components of the DIF-1 signaling cascade do exist and are expressed at more or less similar timings. On the other hand, we were unable to detect DIF-1 production in *A. subglobosum* either biochemically or biologically [38]. Therefore, it is possible that a similar but different substance is produced in *A. subglobosum* and induces a modified version of the polyketide signaling cascade to activate ECM genes and the cellulose synthase required for stalk formation.

The molecular mechanisms of synchronized and rapid spore encapsulation caused by exocytosis of spore-coat materials in the prespore vesicles are relatively well understood. They employ peptides called spore differentiation factor 1 (SDF-1) and SDF-2, which are secreted as precursors from prespore cells and processed by the serine protease-ABC transporters TagB and TagC, respectively [39,40]. The whole process is accelerated by GABA- and MPBD-mediated cascades, the former being triggered by a steroid type SDF (SDF-3) [41]. The accumulation of SDF precursors in the prespore cells is controlled by intracellular cAMP levels *via* protein kinase A [42]. Overlaying *A. subglobosum* orthology and expression data suggested that only the core cascade involving SDF-2 is intact in *A. subglobosum* (Figure 6B); enhancement by the SDF-3-GABA route is probably disabled by the lack of its essential gene, *gadA*. The SDF-1 cascade seems to be hampered by the absence of *tagB*. Considering the small size of the *A. subglobosum* sorus, the synchronous encapsulation of spore precursor cells may not require multiple cascades, although the possibility of functional complementation by paralogous genes still exists.

Overall developmental signalling is compared between the 2 species in Figure 5. We already showed that a sequential gene expression of “prespore” and “prestalk” genes was observed in individual cells [6]. The results presented here suggest that this finding can be extended: In contrast to the developmental process of *D. discoideum* achieved by 2 types of cells in parallel, the developmental program of *A. subglobosum* seems to depend largely on sequential gene expression, which is regulated cell-autonomously, and on a few cell-cell interactions.

## Conclusions

Our genome analysis of *A. subglobosum* revealed the unexpected conservation of *D. discoideum* stalk-specific genes. However, alterations in developmental transcriptomes were extensive. This suggests that non-differentiating species utilize fundamentally different developmental programs, even though their final morphologies appear similar. Since gene losses at the early stages of cell-fate determination must disturb the later developmental processes enormously, they are likely to have been compensated by differential gene regulations.

## Methods

### Cell culture and asexual development

The clonal line of *A. subglobosum* strain LB-1/A1 was described previously (6). A1 cells were grown in shaking HL5 medium at 22°C. For asexual development, the cells were harvested at their early growth phase (1.0–3.0 × 10^6^ cells/mL), washed twice with KK2 buffer (20 mM K_2_HPO_4_/KH_2_PO_4_, pH 6.8), and spread on a cellulose ester membrane (48 mm in diameter) (Advantech) at a density of 2.5 × 10^5^ cells/cm^2^. This was the upper limit for efficient fruiting body formation in *A. subglobosum*. Ten membranes were put on a 20 cm × 20 cm plate of plain agar containing charcoal to enhance development, and incubated at 22°C.

### Genome size determination

Approximately 1 × 10^8^ cells were harvested from the HL5 culture, washed in phosphate-buffered saline (PBS) and pelleted by centrifugation. Their nuclei were prepared using a nuclei extraction kit NE-PER (Pierce), resuspended in 2 mL PBS containing 1 mM EDTA, 200 μg/mL RNase A, and 50 μg/mL propidium iodide, and then analyzed on a FACS Calibur platform (Becton Dickinson) using an excitation wavelength of 488 nm. To ensure single-nucleus measurement, the gate was set using the FLS-A and FL2-W parameters of the doublet discrimination module.

### Chromosome number determination

Approximately 5 × 10^6^ cells were seeded in a 5 cm dish containing acid-washed coverslips and incubated for 2 h in 5 ml HL5 medium to allow cells to adhere. The culture medium was replaced with fresh HL5 containing 33 μM nocotazole. After incubation for 4 h, the coverslips were placed in chilled distilled water for 10 min and fixed for 1 h in ice-cold 3:1 ethanol/glacial acetic acid, followed by 10 min re-fixation in the fresh fixative. The coverslips were air dried and mounted on glass slides in 3 μL DAPI/Vectashield and observed under a wide-field fluorescent microscope using a 100 × 1.4 NA objective.

### Genome sequencing and assembly of *A. subglobosum*

Genomic DNA was extracted from the nuclei of growth phase A1 cells and processed for nucleotide sequencing. We constructed a hybrid *de novo* assembly based on Sanger pair-end whole genome shotgun (WGS) sequences from plasmid clones with a ~3 Kb insert and supplemented with Illumina WGS sequences. The Sanger sequence data were assembled into sequence contigs using PCAP [43] and the subsequently independently assembled Illumina contigs using Platanus [44] were used to extend them and to close gaps between Sanger-based contigs. The transcriptome data (see below) were also used to fill the contig gaps where possible. A fosmid library was also constructed and its 6912 clones were end-sequenced to aid scaffold construction. Some contig gaps were filled by manual walking-in.

### Transcriptome analysis

To construct the growth phase cDNA libraries, mRNA was extracted using Oligotex-dT30 < super > (TAKARA), reverse transcribed, and ligated with (asgl library) or without (asgs library) size fractionation (>1 Kb) to pSPORT1 using the SuperScript Plasmid System with Gateway Technology (Invitrogen) and transformed into *Escherichia coli* DH10B ElectroMax (Invitrogen). For the preparation of developmental RNA, the developing cells were detached from the membranes by incubation in cold PBS containing 5 mM EDTA for 5 min followed by vigorous shaking, and washed twice with cold PBS. A full-length developmental cDNA library (asdv) was constructed from the 3 combined preparations of 20 h cells by the SMART method (TAKARA) using pDNR-LIB as a vector. The inserts of randomly chosen clones from these 3 cDNA libraries were sequenced from both ends using an ABI 3730 DNA Analyzer (Applied Biosystems). The obtained EST data were assembled by the CAP3 program to obtain non-redundant sequences. We selected and re-sequenced 700 clones with an unfilled internal sequence to generate high quality cDNA sequences.

For mRNA massive sequencing, we combined 6 independent preparations of total RNA from 0, 8, 16, and 24 h of development. The cDNA templates for Solexa sequencing were synthesized using an mRNA-Seq RNA Sample Prep Kit (Illumina) according to the manufacturer’s instructions. The sequence data were assembled using ABySS [45] and mapped to the genome contigs using the exonerate assembly program [46].

### Gene model construction

*A. subglobosum* gene models were constructed by the following 3 methods. 1) Acyto_CDS: the cDNA sequences and CAP4 assembly of ESTs were aligned to the genome contigs and those with an identity ≥ 95% and coverage ≥ 80% were selected. Where it was appropriate, the forward and reverse sequences of each singlet were joined. The longest open reading frames starting with the initiation codon were adopted. 2) Dicty_Pept: the translated *A. subglobosum* genome sequences homologous to *D. discoideum* protein sequences at a similarity ≥ 30% and coverage ≥ 50% were extracted and joined where appropriate. 3) *Ab initio* prediction: a gene prediction program based on dicodon analysis [15] was used employing the real transcript information obtained here. The final gene models were constructed by unifying the above 3 models and, in part, by manual curation.

### Genome information and gene models of other dictyostelid species

The genome sequences and gene models of *D. discoideum*, *D. purpureum*, *P. pallidum*, and *D. fasciculatum* were downloaded from dictyBase [27]. For *D. discoideum,* the genes located on the duplicated region of chromosome 2 [10] were eliminated. *D. discoideum* “developmental genes” were extracted from published reports summarized in the Dicty Stock Center website [47].

### Gene orthology and family assignment

Orthologous gene pairs were determined between 2 species by the bidirectional best hit approach setting the blastp threshold to an e-value of 1E-10. Genes of non-orthologous hits were regarded as paralogs in each species. We used OrthoMCL [48] to cluster the proteins of *D. discoideum* and manually supplemented the results using information from the dictyBase gene list and reports by Sucgang et al. [11] and Heidel et al. [12]*.* Orthologous genes in other species and their paralogs were assigned to the same gene family.

### Transcriptome comparison

The mRNAseq data of *A. subglobosum* obtained as the mean of 6 biological replicates, excluding contaminating rRNA sequences, were converted to reads per kilobase per million as in the case of *D. discoideum*. For the downloaded *D. discoideum* data of Parikh et al. [13], those from 0, 8, 16, and 24 h were extracted and the mean of 2 biological replicates was obtained. To normalize the data of the 2 species, each value was multiplied by [10^7^/the sum of the relative expression levels for each time point of each species]. Genes that were not expressed throughout development were eliminated, and all remaining data of the 2 species were combined. K-means clustering was performed using Orange software [49] with distance measure, Pearson correlation, initialization, random, and restart 100 times. Cluster number 8 was employed after trials using larger and smaller numbers.

### Availability of supporting data

The data sets supporting the results of this article were deposited to DDBJ under project ID PRJDG1513. Their accessions are HY448297-HY508708 for 60412 EST, BAUZ01000001-BAUZ01000371 for 371 WGS and DF837573-DF83768 for 106 CON (Contiguous sequence) entries. CON entries include 11687 CDS loci (locus_tag: SAMD00019534_000010- SAMD00019534_126860; protein_id: GAM116827-GAM29510). Protein sequences of gene models are also supplied in Additional file 3.

## Additional files

Additional file 1: Figure S1. Size distribution of analyzed *A. subglobosum* supercontigs. **Figure S2.** Properties of *A. subglobosum* coding genes deduced from cDNA and EST analyses. **Figure S3.** Results of *A. subglobosum* gene prediction. **Figure S4.** Gene family distributions in *D. discoideum.* **Figure S5.** Lineage-specific family expansions. **Figure S6.** A phylogenetic tree of the ABC transporter B family, serine protease family proteins. **Figure S7.** Statistics of mRNAseq results. **Figure S8.** K-means clustering of developmental transcriptomes. **Figure S9.** Differential expression of orthologous gene pairs between *A. subglobosum* and *D. discoideum*. Additional file 2: Table S1. Comparison of codon frequencies. **Table S2.** Homology and Expression data of *A. subglobosum* genes. **Table S3.** Downloaded and processed *D. discoideum* data. **Table S4.** *D. discoideum* developmental genes with altered expression in *A. subglobosum* (shown by DDB_G ID). Additional file 3:
**Protein sequences of gene models used in the present study.**
