# Supplementary material for: Comparative genome and transcriptome analyses of the social amoeba Acytostelium subglobosum that accomplishes multicellular development without germ-soma differentiation
Source: BMC Genomics. 2015 Feb 14;16(1):80. doi: 10.1186/s12864-015-1278-x (PMC4334915; doi:10.1186/s12864-015-1278-x)
Supplement: Additional file 1: Figure S1. — Size distribution of analyzed A. subglobosum supercontigs. Figure S2. Properties of A. subglobosum coding genes deduced from cDNA and EST analyses. Figure S3. Results of A. subglobosum gene prediction. Figure S4. Gene family distributions in D. discoideum. Figure S5. Lineage-specific family expansions. Figure S6. A phylogenetic tree of the ABC transporter B family, serine protease family proteins. Figure S7. Statistics of mRNAseq results. Figure S8. K-means clustering of developmental transcriptomes. Figure S9. Differential expression of orthologous gene pairs between A. subglobosum and D. discoideum. [file 12864_2015_1278_MOESM1_ESM.pdf]

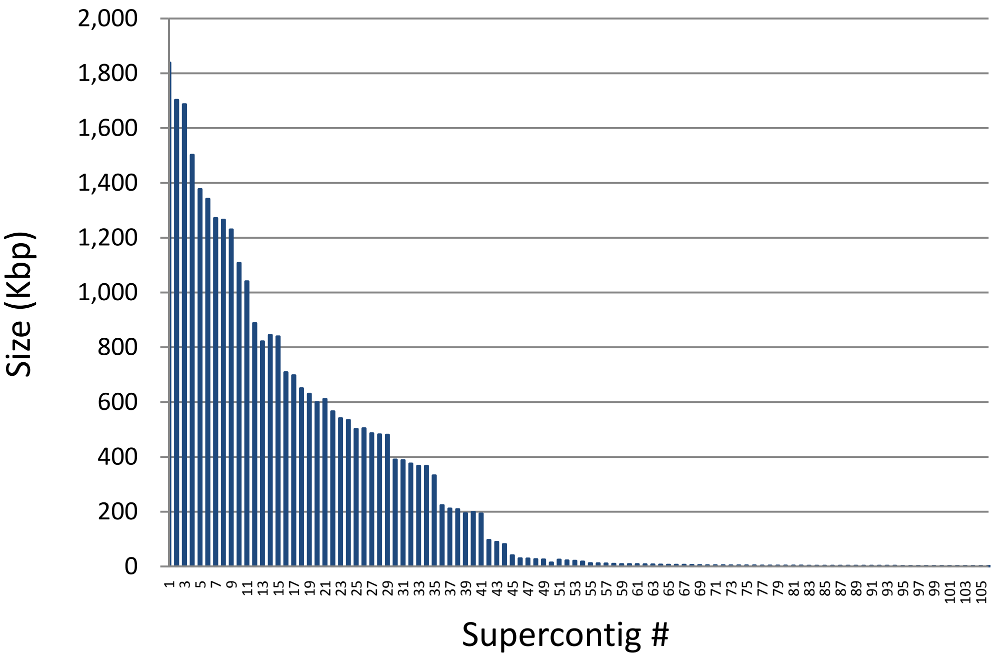

Supplementary Figure 1. Size distribution of analyzed *A. subglobosum* supercontigs. At the current stage of genome assembly, identification of centromere and telomere sequences was not possible.

(A)

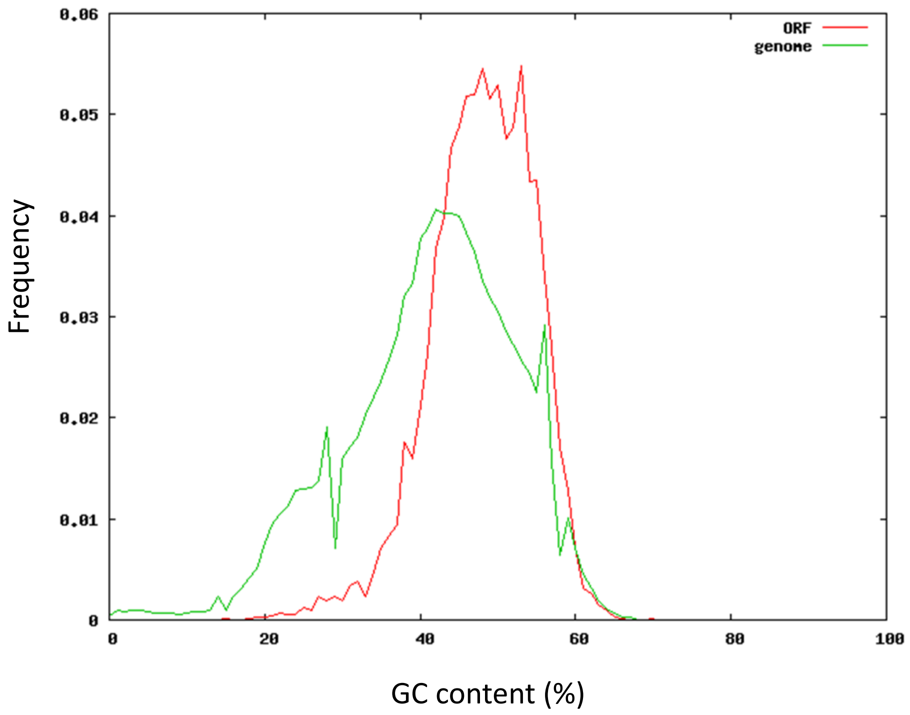

(B)

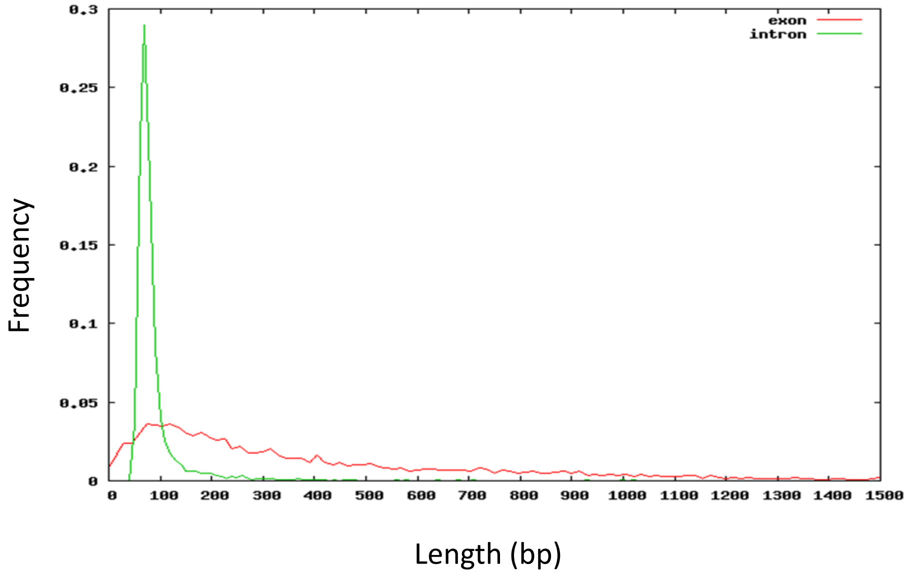

Supplementary Figure 2. Properties of *A. subglobosum* coding genes deduced from cDNA and EST analyses. **A**: (G+C) content distribution of ORF (red) and genome (green) are shown in 200 bp-windows. **B**: Size distribution of exon (red) and intron (green). The intron peaks at 70 bp.

(A)

(B)

(C)

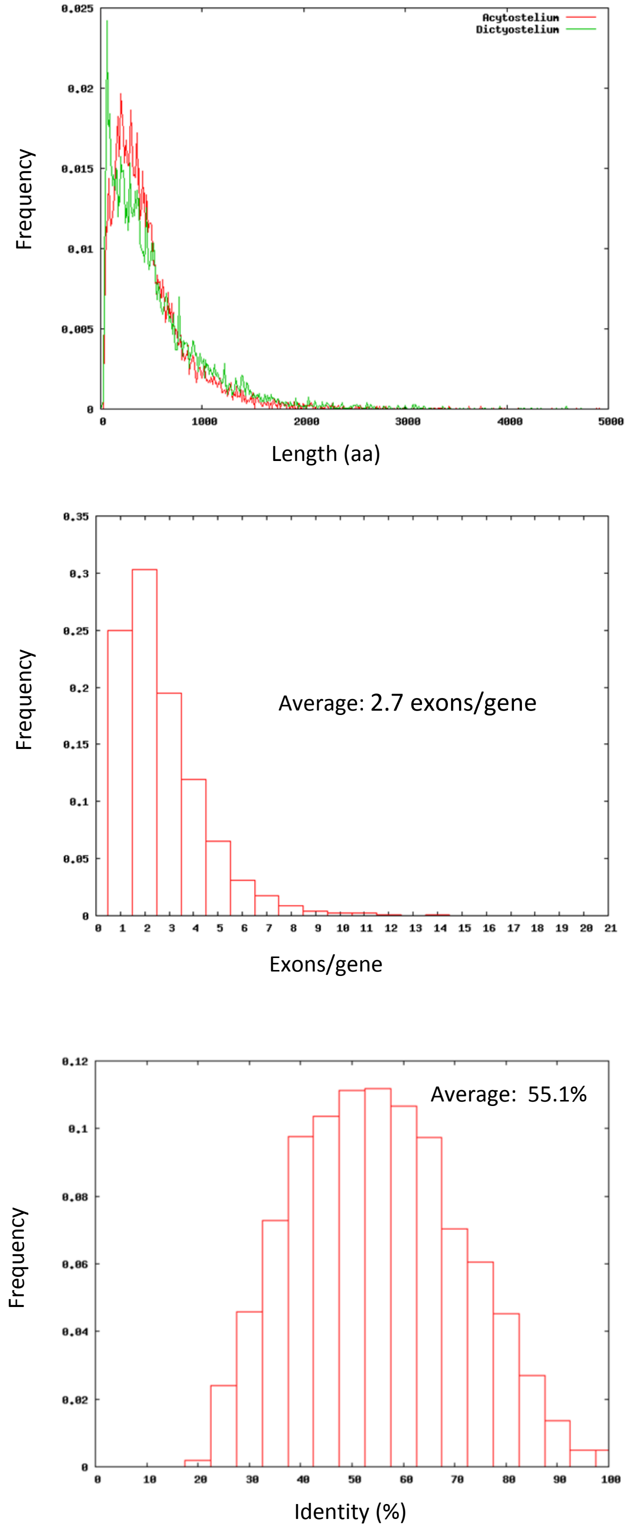

Supplementary Figure 3. Results of *A. subglobosum* gene prediction. **A**: Gene length were compared in terms of amino acid length between *A. subglobosum* (red) and *D. discoideum* (green). **B**: Distribution of exon number per gene is shown. **C**: Distribution of ortholog identity is shown. Orthologous gene pairs between *A. subglobosum* and *D. discoideum* gene models were obtained as blastp reciprocal best hits and coverage >= 60%.

(A)

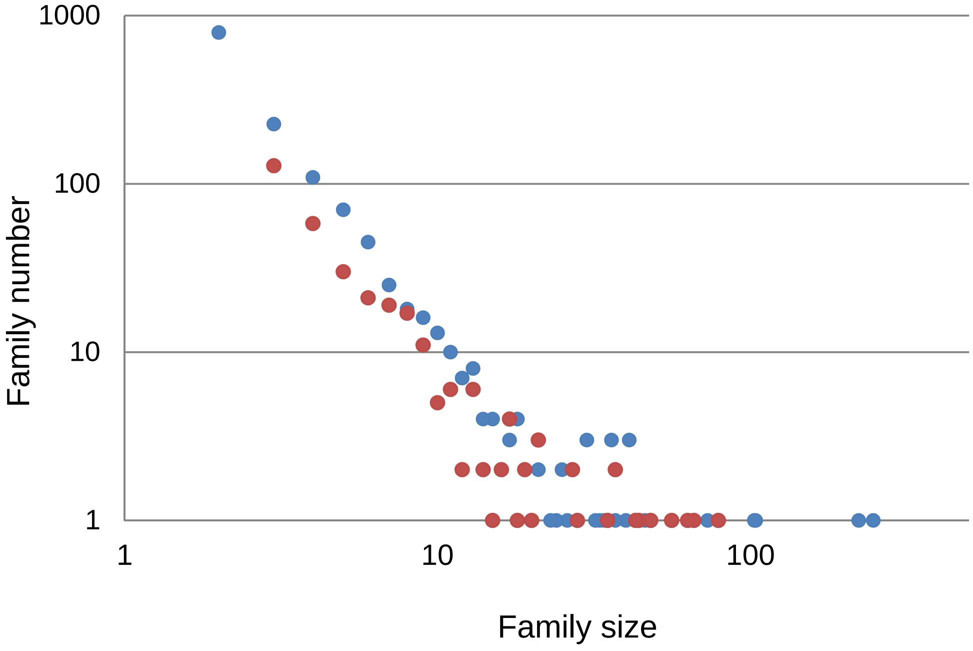

(B)

Family data	Max size	Average size (>=3)	Family number (size >=3)
**orthoMCLDB**	247	7.69	597 (4595 genes)
**Reference 10**	79	7.07	332 (2350 genes)

Supplementary Figure 4. Gene family distributions in *D. discoideum*. **A**: Numbers of families against family size indicated on the abscissa were plotted for the downloaded data from OrthoMCL (blue) and Eichinger et al. [10] (red). **B**: Two datasets were summarized.

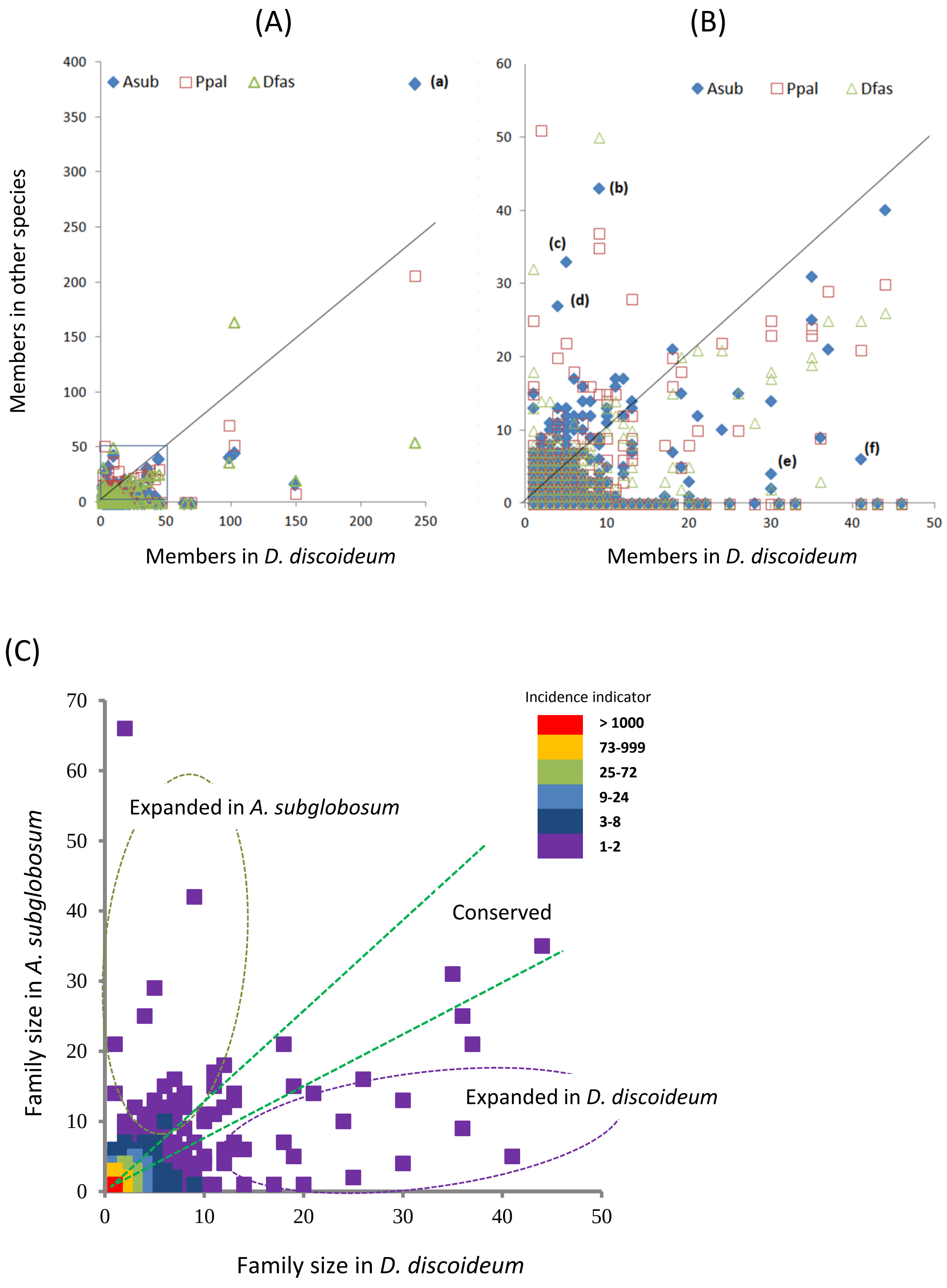

Supplementary Figure 5. Lineage-specific family expansions. **A**: Number of family members in each species are plotted against those in *D. discoideum.* Solid line indicates Y = X. **B**: Only families smaller than 50 members in *D. discoideum* are shown. Families marked by (a) to (F) correspond to those listed in Table 2. **C**: *A. subglobosum* data were extracted from (B) and numbers of families were color indicated.

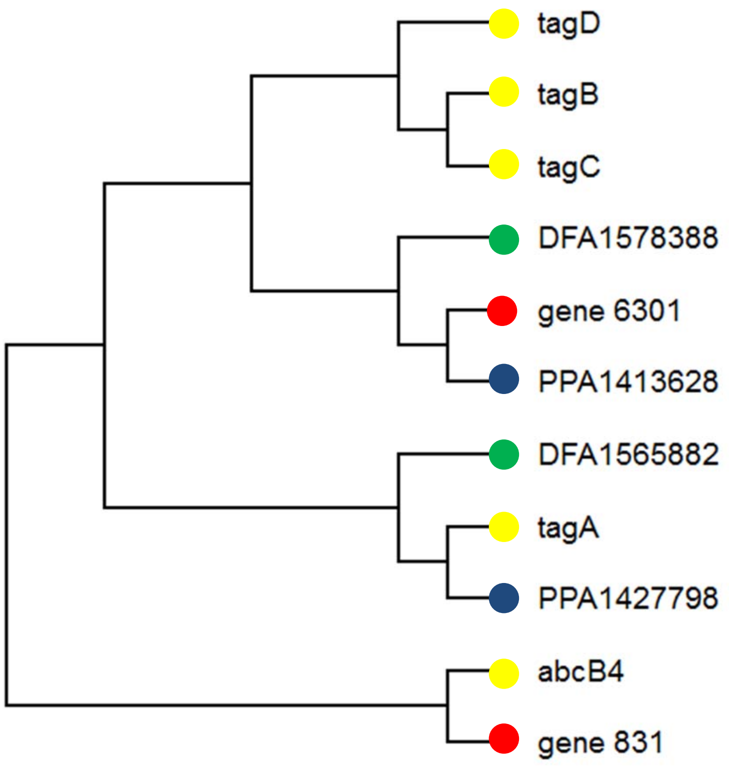

Supplementary Figure 6. A phylogenetic tree of the ABC transporter B family, serine protease (OG5_134947) family proteins. Yellow, red, blue, and green dots indicate *D. discoideum, A. subglobosum, P. pallidum,* and *D. fasciculatum* proteins, respectively. *A. subglobosum* gene 831, which is the closest homolog to *tagA* of this species, and its *D. discoideum* ortholog abcB4 do not contain the serine protease domain and constitute an outgroup.

A: mRNA-seq data and direct mapping

Sample		0 h	8 h	16 h	24 h	Total
Raw data	Tag	54,724,646	52,032,738	54,724,676	56,306,952	217,789,012
	Extension (Gbp)	5.7	5.5	5.7	5.9	22.8
Trimmed data	Tags >= 75bp	37,463,896	36,430,504	38,280,426	38,973,350	151,148,176
	Extension (Gbp)	4.4	4.3	4.5	4.5	17.7
mapped data	all	45.5%	45.8%	44.7%	44.9%	45.2%
	unique	41.4%	42.2%	42.6%	43.0%	42.3%

B: Assembly and mapping results

Condition	Contigs	ratio (%)	Genome sites
Total contig (>= 300 bp)	17,954	N/A	N/A
Identity >= 95%, Coverage >= 50%	17,823	99.3%	17,907
Identity >= 97.5%, Coverage >= 95%	16,704	93.0%	16,780
Exact match	13,735	76.5%	13,790

Supplementary Figure 7. Statistics of mRNAseq results.

(A)

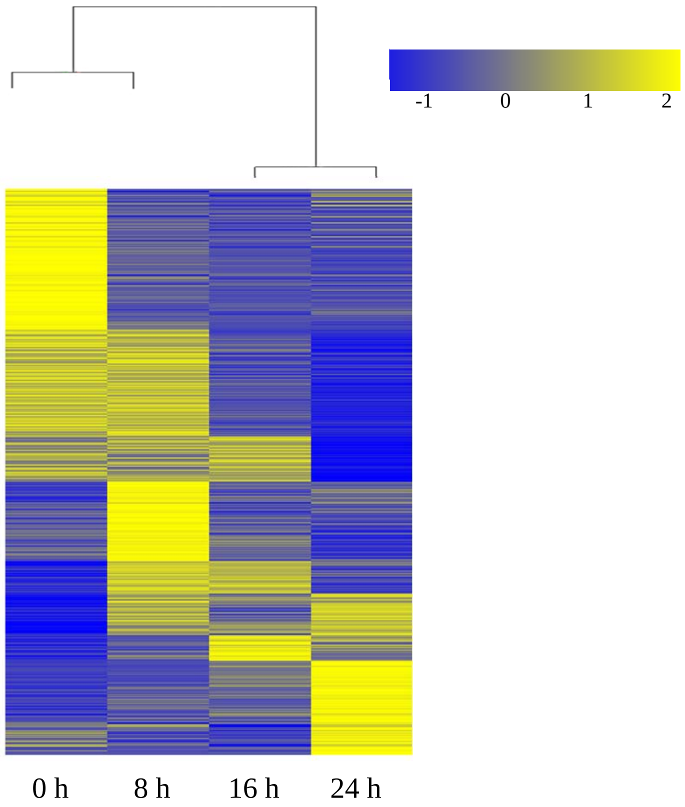

(B)

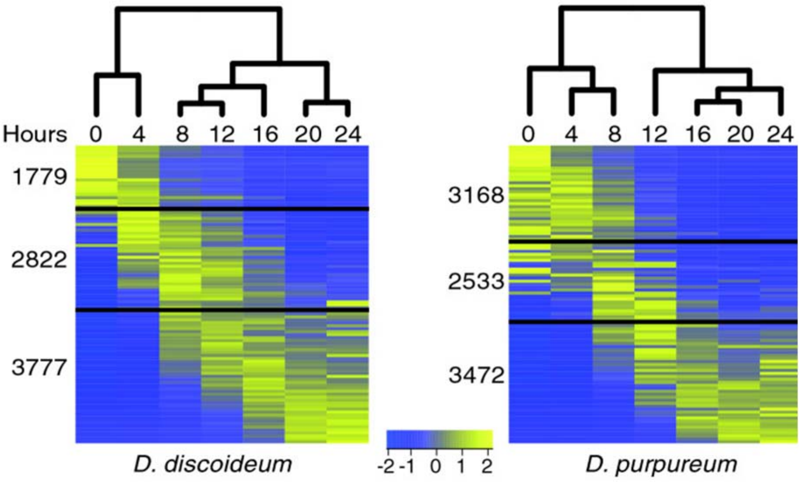

Supplementary Figure 8. K-means clustering of developmental transcriptomes. **A**: The result of *A. subglobosum* transcriptome analysis is shown. **B**: *D. discoideum* (left) and *D. purpureum* (right) data from Parikh et al [13] are shown for comparison.

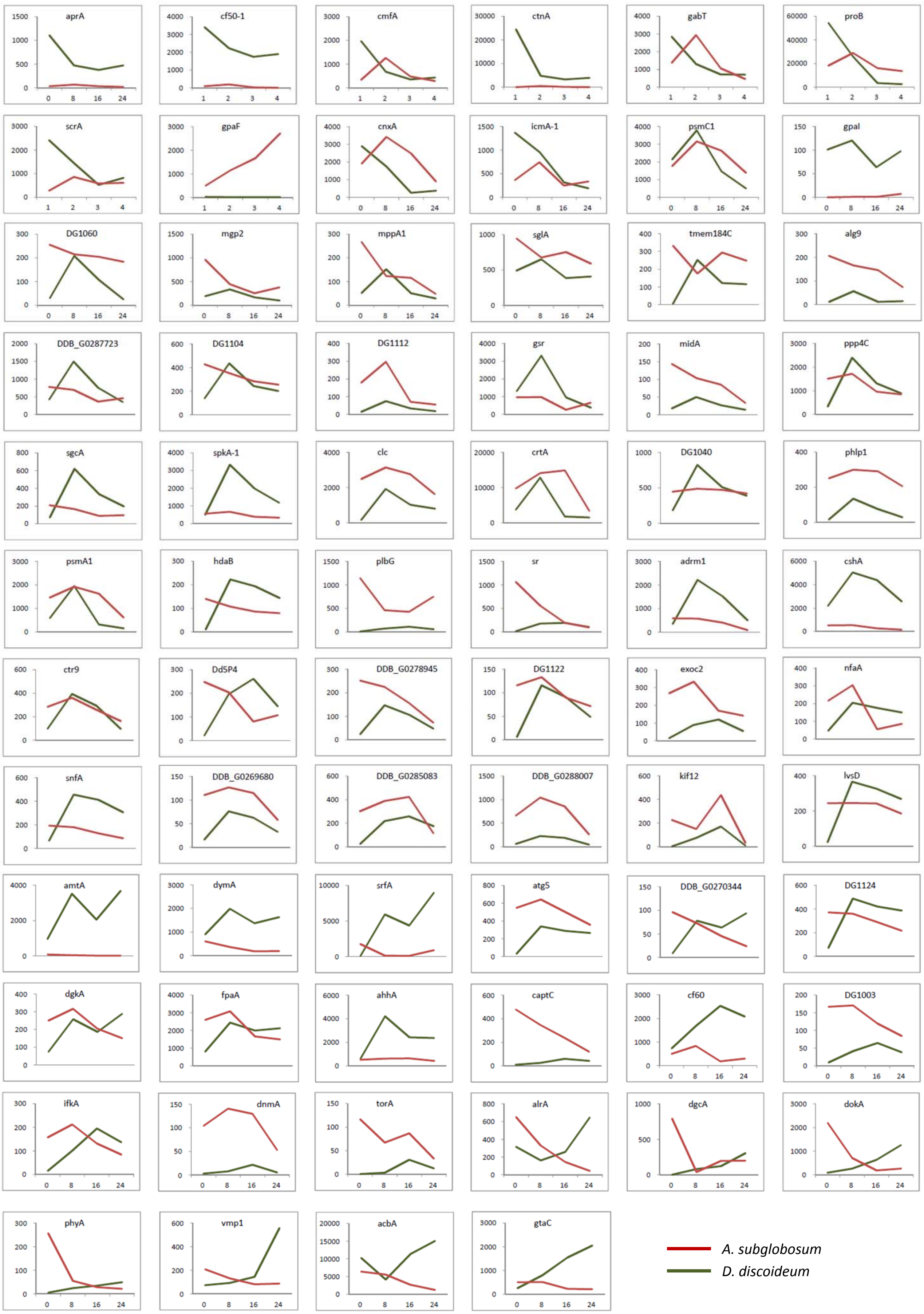

Supplementary Figure 9. Differential expression of orthologous gene pairs between *A. subglobosum* and *D. discoideum*. Only genes listed in Table 4 are shown. Red and green lines represent expressions of *A. subglobosum* and *D. discoideum*, respectively.
